# Vagal Reactions during Cryoballoon-Based Pulmonary Vein Isolation: A Clue for Autonomic Nervous System Modulation?

**DOI:** 10.1155/2016/7286074

**Published:** 2016-05-03

**Authors:** Michaël Peyrol, Jérémie Barraud, Linda Koutbi, Baptiste Maille, Lory Trevisan, Elisa Martinez, Samuel Lévy, Franck Paganelli, Frederic Franceschi

**Affiliations:** ^1^Service de Cardiologie, Centre Hospitalier Universitaire de Marseille, Hôpital Nord, Aix-Marseille Université, 13915 Marseille Cedex 20, France; ^2^APHM, Department of Cardiology, Timone University Hospital, 13385 Marseille Cedex 05, France; ^3^Aix-Marseille Université, 13284 Marseille Cedex 07, France

## Abstract

Although paroxysmal atrial fibrillation (AF) is known to be initiated by rapid firing of pulmonary veins (PV) and non-PV triggers, the crucial role of cardiac autonomic nervous system (ANS) in the initiation and maintenance of AF has long been appreciated in both experimental and clinical studies. The cardiac intrinsic ANS is composed of ganglionated plexi (GPs), located close to the left atrium-pulmonary vein junctions and a vast network of interconnecting neurons. Ablation strategies aiming for complete PV isolation (PVI) remain the cornerstone of AF ablation procedures. However, several observational studies and few randomized studies have suggested that GP ablation, as an adjunctive strategy, might achieve better clinical outcomes in patients undergoing radiofrequency-based PVI for both paroxysmal and nonparoxysmal AF. In these patients, vagal reactions (VR) such as vagally mediated bradycardia or asystole are thought to reflect intrinsic cardiac ANS modulation and/or denervation. Vagal reactions occurring during cryoballoon- (CB-) based PVI have been previously reported; however, little is known on resulting ANS modulation and/or prevalence and significance of vagal reactions during PVI with the CB technique. We conducted a review of prevalence, putative mechanisms, and significance of VR during CB-based PVI.

## 1. Introduction

Paroxysmal atrial fibrillation (AF) is known to be initiated by rapid firing of pulmonary veins (PVs) and non-PV triggers [1, 2]. In parallel, the crucial role of cardiac autonomic nervous system (ANS) in the initiation and maintenance of atrial fibrillation (AF), especially paroxysmal AF, has long been appreciated in both experimental and clinical studies [3–8]. Indeed, stimulation of cardiac ANS may induce AF in both animals and humans [3–8]. Conversely, parasympathetic nerve denervation has been demonstrated to prevent induction of AF in canine study [9, 10].

The cardiac intrinsic ANS is composed of ganglionated plexi (GPs) and a vast network of interconnecting neurons [7, 11]. The major left atria GPs are located in epicardial fat pads at 4 preferential locations close to the left atrium-pulmonary vein (PV) junctions: superior left GP, inferior left GP, anterior right GP, and inferior right GP [7, 11]. The ganglionated plexi act as an integration and interconnection system between the cardiac extrinsic ANS nerves, originating from the central ANS and reaching the heart through the mediastinum, and the rest of the intrinsic cardiac ANS, the so-called “atrial neural network” [7, 11].

Ablation strategies targeting the PV and aiming for complete PV isolation (PVI) remain the cornerstone of AF ablation procedures [12]. Radiofrequency is the conventional energy source used by cardiac electrophysiologists to achieve PVI [12]. However, the cryoballoon (CB) technique has also been validated as a safe and valuable tool in order to achieve PVI and represents an alternative to radiofrequency [12–18].

Several observational studies and few randomized studies have suggested that GP ablation, as an adjunctive strategy, might achieve better clinical outcomes in patients undergoing radiofrequency-based PVI for both paroxysmal and nonparoxysmal AF [19–22]. In these patients, vagal reactions (VR) such as vagally mediated bradycardia or asystole are thought to reflect intrinsic cardiac ANS modulation and/or denervation [19–22]. Heart rate variability (HRV) has been suggested as a simple and effective noninvasive tool for evaluation of ANS activity [6, 8, 19, 22]. Reduction of HRV has been proposed as an indicator for cardiac ANS modulation in patients undergoing PV radiofrequency ablation [6, 8, 19, 22].

Vagal reactions occurring during CB-based PVI have been previously reported; however, little is known on resulting ANS modulation and/or prevalence and significance of VR during PVI with the CB technique. We conducted a review of prevalence, putative mechanisms, and significance of VR during CB-based PVI.

## 2. Cardiac Autonomic Denervation during Radiofrequency Pulmonary Vein Ablation

Ganglionated plexi ablation aiming for modulation/denervation of the intrinsic cardiac ANS has been proposed as an adjunctive strategy for radiofrequency catheter ablation of paroxysmal and nonparoxysmal AF [19–22]. Ganglionated plexi locations may be identified during AF ablation by endocardial high frequency electric stimulation at sites exhibiting VR (i.e., bradycardia below 40 bpm or asystole) [19, 20, 22]. Thereafter, these sites, always showing complex fractionated atrial activity during AF, are targeted during endocardial radiofrequency catheter ablation with abolishment of VR as the ablation endpoint [12, 19–22]. Anatomically guided ablation of GP has been demonstrated to be noninferior to the high frequency pacing strategy, probably because cardiac GPs exhibit only small variations in location [11]. In fact, GP ablation often occurs during wide circumferential PV ablation as a “collateral damage” of procedures aiming for PVI with large antral linear lesions [19, 20, 23]. In an observational study, Pappone et al. reported VR in 102/297 patients (34.3%) during wide circumferential PVI [19]. They indicated that VR mostly occurred during radiofrequency application at the cranial junction of the left superior PV and the left atrium (in 95% of patients with VR) and at the posteroinferior junction between the left inferior PV and left atrium (71 patients, 70%) [19]. These sites are known to be the locations of 2 of 4 major left atrial GPs [7, 11]. Additionally, intrinsic cardiac ANS denervation, assessed by reduction of HRV, was observed in up to one-third of patients undergoing wide circumferential PVI but was limited for a 3-month period following index procedure [19]. However, when RF lesions were created using an open irrigation tip catheter, cardiac ANS modulation was maintained 1 year after the index procedure [24, 25]. In these aforementioned studies, cardiac ANS modulation was associated with lower AF recurrence [19, 24, 25]. When added to PVI, RF lesions applied to the GPs significantly improved outcomes in randomized study by Katritsis et al. [21]. After a 2-year follow-up, freedom from AF or atrial tachycardia was of 56% and 74% in PVI alone and PVI + GP ablation groups, respectively [21]. Conversely, GP ablation alone conferred a worse outcome than PVI alone, where it was shown that freedom from AF or atrial tachycardia was 48% and 56% in GP ablation and PVI groups, respectively (*p* = 0.001 for comparison between groups) [21].

## 3. Cardiac Autonomic Denervation during Cryoballoon-Based Pulmonary Vein Ablation

The CB technique targets the PVs and adjacent myocardium with the aim of obtaining a complete PVI [12–18]. It is achieved for a very high proportion of patients after a single procedure, conferring a very acceptable outcome [13–18].

Little is known regarding intrinsic cardiac ANS modulation during CB-based PVI. Oswald et al. were the first to investigate the effect on ANS when applying cryoenergy at the PV ostia, although it should be mentioned that the PVIs were performed with the first generation CB (Arctic Front, Cryocath, Montreal, Canada) [26]. Similarly to RF studies, the authors used HRV as a marker of ANS modulation. Although the study sample was small (14 paroxysmal AF patients), they demonstrated that AF cryoablation was associated with significant ANS modulation. Indeed, 12 out of 14 patients (86%) presented with significant decrease in HRV after the cryoablation procedure. Five patients (36%) required temporary pacing for symptomatic intraprocedural bradycardia <40 bpm or asystole but the 3 largest HRV reductions occurred in patients without VR during the procedure. Interestingly the authors reported that the 2 patients that did not show HRV decrease were free of AF recurrence thereafter. Five patients out of 12 with decrease in HRV (41.7%) experienced AF recurrence during follow-up. The authors hypothesized that VR might be induced by either stretch of the left atrial tissue, direct damage to cardiac nerve tissue during the thawing phase, or hyperemia at the CB-tissue interface.

The second generation of CB (Arctic Front Advance*™*, Medtronic) has been redesigned to enhance lesion quality. The goal was to create transmural contiguous lesions in order to achieve durable PVI. The number of refrigerant injection ports has been increased from 4 to 8 with a more distal orientation, resulting in higher refrigerant flow (for Arctic Front Advance 28 mm) that improves balloon cooling uniformity with lower temperature at the CB surface. Compared to the first generation CB, Arctic Front Advance CB has been demonstrated to increase single-shot PVI rate and decrease time to PVI as well as having shorter procedure and radiation exposure times [15, 16]. Importantly, freedom from AF at mid-term follow-up appeared higher with the Arctic Front Advance CB [17, 18]. Thanks to these technical features, VR might be expected to occur more often with the second generation of CB.

Yorgun et al. specifically studied the impact of VR during PV cryoablation on clinical outcomes in 145 patients with paroxysmal (81%) or persistent drug-refractory AF treated with the second generation CB [27]. After a 17-month follow-up period, 119/145 patients (82.1%) were free from AF recurrence. Notably, they reported a high prevalence of VR (40.7%), characterized as significant bradycardia and/or hypotension during CB application at the PVs. Intravenous atropine administration, to treat symptomatic heart rate drop below 40 bpm or pauses, was required in up to 33.1% of patients. Interestingly, VR or the need of atropine administration was more frequent in patients without AF recurrence after the end of follow-up (46.2% versus 15.4%, *p* = 0.004 and 38.7% versus 7.7%, *p* = 0.002, resp.). Using a univariate Cox proportional hazard regression analysis, Yorgun et al. found that VR and requirement of atropine administration or cardiac pacing for severe bradycardia decreased the risk of AF recurrence. Using a multivariate Cox proportional hazard regression analysis, the authors demonstrated that the need of atropine administration was an independent predictor of AF recurrence in patients undergoing PVI with the CB technique (HR: 0.064; 95% CI: 0.008–0.48, *p* = 0.008) while VR was not (HR: 2.59; 95% CI: 0.56–12.05, *p* = 0.225) [27]. Otherwise, in their discussion, Yorgun et al. hypothesized that the use of the bigger 28 mm CB might generate more VR compared to the 23 mm CB due to more antral cryolesions location. Finally, unanswered question underlined by the authors is whether VR, as a clue for ANS modification, might be considered as a potential end-point of CB-based PVI procedures or only represents a marker of collateral damage during PV cryoablation.

Aytemir et al. recently tried to define the predictive factors of late AF recurrence with the current CB when compared to the first generation CB [28]. They reported a series of 306 consecutive patients (mean age of 55.3 ± 10 years) presenting with drug-refractory symptomatic paroxysmal (80.7%) or persistent AF. They underwent PVI attempt using either the first generation CB (*n* = 197) or the second generation CB (*n* = 109 patients). Main conclusion was that the use of the second generation CB resulted in lower late AF recurrence rate. They also demonstrated that both intraprocedural VR and second generation CB use were associated with fewer late AF recurrences. Furthermore, it was found that VR were more frequently observed with the second generation CB compared with the first generation CB (50.4% versus 38.1%; *p* = 0.036). Also, systematic use of the largest 28 mm CB that resulted in more antral CB positioning compared to the 23 mm model might have explained, by itself, the higher prevalence of VR. Multivariate Cox proportional hazard regression analysis related to the late AF recurrence following single cryoablation identified left atrium diameter, early recurrence, and Artic Front Advance CB use as the only predictive factors of late AF recurrence. Vagal reactions tended to be predictive for lower late AF recurrence, but did not reach statistical significance (HR: 0.574; 95% CI: 0.335–1.002, *p* = 0.055).

In the three aforementioned studies, several findings are reported. First, the occurrence of VR during CB-based PVI is reported to be frequent, ranging from 36% to 50%, which is comparable to RF studies, and higher with the second generation CB [19, 26–28]. Secondly, VR during PV cryoablation with the first generation CB is considered as a marker of intrinsic ANS modification but analysis of postablation HRV showed that this latter lasts no more than 3 months [26]. It should be mentioned that the second generation CB, resulting in more extensive myocardial injury, might have a more sustained impact on postablation HRV. Finally, intraprocedural VR are associated with lower rates of AF recurrence [24–26]. Nevertheless, some data are lacking to better characterize VR during PVI with the CB technique. Also, little is known regarding clinical characteristics of patients presenting VR during PV cryoablation. Similarly, data on HRV following PV cryoablation with the second generation CG or putative mechanisms of improved outcome for patients experiencing VR during CB-based PVI are lacking.

**Figure 1:**
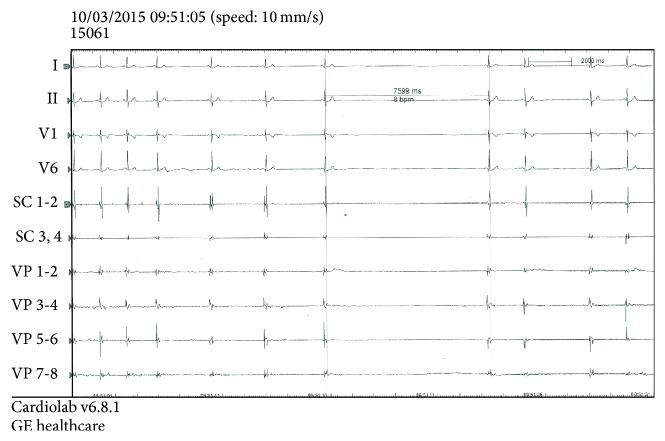
Vagally mediated asystole during thawing phase of the cryoballoon after a 180-second application at the left superior pulmonary vein ostium in a patient with drug-refractory paroxysmal AF. Note that the progressive sinus rhythm rate decreases followed by a 7.6-second asystole. Sinus rhythm resumed thereafter. Time to pulmonary vein isolation was 26 seconds and CB temperature at PVI time was −27°C. Minimal CB temperature reached was −52°C. After 18 months of follow-up, the patient was free from any atrial arrhythmia recurrence. SC: coronary sinus; VP: pulmonary vein.

In our practice, we currently use the CB technique for PVI in paroxysmal AF patients for nearly 8 years. In our experience of 980 PV cryoablation procedures performed in our institution (280 and 700 procedures with the first and second generation of CB, resp.) and although we did not specifically investigate the role of VR during PV cryoablation as a predictive factor for AF recurrence, we found a lower prevalence of vagally mediated bradycardia during CB application, about 5 to 10% (unpublished data). Even lower prevalence of VR was observed with the first generation CB compared with the second generation CB. Vagally mediated bradycardia was transient, was self-limited, and required exceptionally temporary pacing or atropine administration. Similar to previous studies, we noticed VR during the CB thawing phase or at time of CB deflation [26–28]. Vagally-mediated bradycardia was systematically related to sinus bradycardia or sinus arrest, exclusively following CB application at the left-sided PVs (Figure 1). We never observed transient atrioventricular block and/or VR following cryoapplication at the right-sided PVs. This fact may be explained by the nervous connections between the left-sided GPs and the sinus node, whereas right-sided GPs are mainly connected with the atrioventricular node [7, 11]. Rarely, VR might occur during CB placement at the PV ostium and during PV occlusion attempt. This maneuver may result in significant left atrial stretch, known to be a putative mechanism for VR [26]. More importantly, we pointed out that VR was reproducible after “bonus” cryoapplication, that is, following additional CB application after achievement of acute PVI. Vagal reactions were neither attenuated nor enhanced after successive CB applications in our experience. Therefore, we think that the reproducibility of VR during “bonus” cryoapplication strongly rejects the hypothesis of intrinsic ANS modification/denervation during PV cryoablation. We believe that VR during PV cryoablation should rather be considered as a marker of intrinsic cardiac ANS stimulation rather than a marker of intrinsic cardiac ANS modulation/denervation. In the same line, RF studies have shown progressive reduction of intensity of VR during CPVI with successful ANS denervation [19–23]. On the other hand, we think that VR during CB-based PVI is a highly specific marker of the transmurality of the cryolesion comparable to that reported with phrenic nerve palsy at the right-sided PVs [29]. Finally, ANS reinnervation might be a putative mechanism for both recovery of ANS activity and recurrence of atrial arrhythmia [30, 31].

## 4. Conclusion

Vagal reactions during CB-based PVI are not infrequent. Some authors consider VR during PV cryoablation as a marker of ANS modification. Due to the temporary effect of PV cryoablation on ANS activity with the first generation CB (assessed by analysis of HRV with gradual recovery of normal HRV at 3 months), questions remain regarding the mechanism of improved outcome for patients experiencing VR during CB-based PVI. Larger studies conducted with the second generation CB and achieving long-term follow-up are needed to validate this hypothesis and to confirm the putative mechanisms.
